# Robust Bisulfite‐Free Single‐Molecule Real‐Time Sequencing of Methyldeoxycytidine Based on a Novel hpTet3 Enzyme

**DOI:** 10.1002/anie.202418500

**Published:** 2024-11-25

**Authors:** Hanife Sahin, Raheleh Salehi, Shariful Islam, Markus Müller, Pascal Giehr, Thomas Carell

**Affiliations:** ^1^ Center for Nucleic Acid Therapies at the Department of Chemistry Institute for Chemical Epigenetics Ludwig-Maximilians-Universität München Butenandtstr. 5-13 81377 München Germany

**Keywords:** Epigenetics, SMRTseq, Tet enzymes, recurrent neural network, methyldeoxycytidine

## Abstract

In addition to the four canonical nucleosides dA, dG, dC and T, genomic DNA contains the additional base 5‐methyldeoxycytidine (mdC). The presence of this methylated cytidine nucleoside in promoter regions or gene bodies significantly affects the transcriptional activity of the corresponding gene. Consequently, the methylation patterns of genes are crucial for either silencing or activating genes. Sequencing the positions of mdC in the genome is therefore of paramount importance for early cancer diagnostics as it helps determine incorrect gene expression. Currently, the bisulfite method is the gold standard for mdC‐sequencing. However, this method has the drawback that the majority of the input DNA is degraded during the bisulfite treatment. Additionally, bisulfite sequencing is prone to errors. Here, we report a benign, bisulfite‐free mdC sequencing method termed EMox‐seq, which is based on third‐generation single‐molecule SMRT sequencing. The foundation of this technology is a new Tet3 enzyme that efficiently oxidizes mdCs to 5‐carboxycytidine (cadC). In turn, cadC provides an excellent readout by SMRT sequencing using specially trained AI‐based algorithms.

## Graphical abstract

EMox‐seq (enzymatic methylcytidine oxidation sequencing) enables bisulfite‐free, single molecule sequencing of DNA methylation patterns at high precision with just a single enzymatic treatment step, using a high‐performance Tet3 catalytic domain.

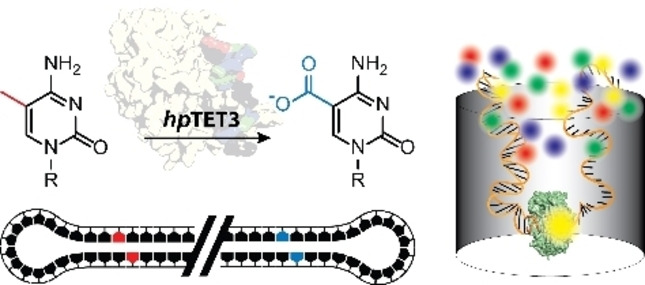

The presence of the fifth nucleoside 5‐methyldeoxycytidine (mdC) in either promoter regions or the gene body, influences the transcriptional state of the corresponding gene.^[1]^ Typically, the presence of mdC in promoter regions silences the gene, while unmethylated promoters indicate more active transcription. Identifying mdC within genes allows characterization of the transcriptional state of the gene of interest, which is crucial for identifying and characterizing tumor cells.[^2^, ^3^] In tumor cells, oncogenes are often wrongly activated while tumor suppressor genes are aberrantly switched off. Therefore, sequencing mdC with minimal input material is highly desired to establish a new tumor diagnostic area, called liquid biopsy.^[4]^

To date, mdC sequencing is predominantly performed using bisulfite treatment (Figure 1a). Genomic DNA exposed to bisulfite at >60 °C converts all non‐methylated cytidines into uracil, while mdC remains intact. Following PCR and sequencing, comparing the obtained reads with a reference genome allows the determination of mdC positions in the genome. However, a significant issue with this method is that much of the genomic input DNA does not survive the harsh bisulfite treatment conditions due to extensive DNA fragmentation. This limitation is mitigated by extensive PCR‐based amplification of the non‐degraded DNA. Another drawback is that the bisulfite sequencing protocol is cumbersome and error prone. Milder methods under development, like EM‐seq, utilize the deaminating enzyme APOBEC3 A (A3 A), which also deaminates dC to dU.^[5]^ However, deamination of all dC bases to dU reduces the genome complexity from a four letter code to a three nucleobase code (dA, dG and dU, plus the remaining mdC), making sequence mapping challenging, especially for repetitive elements.

**Figure 1:**
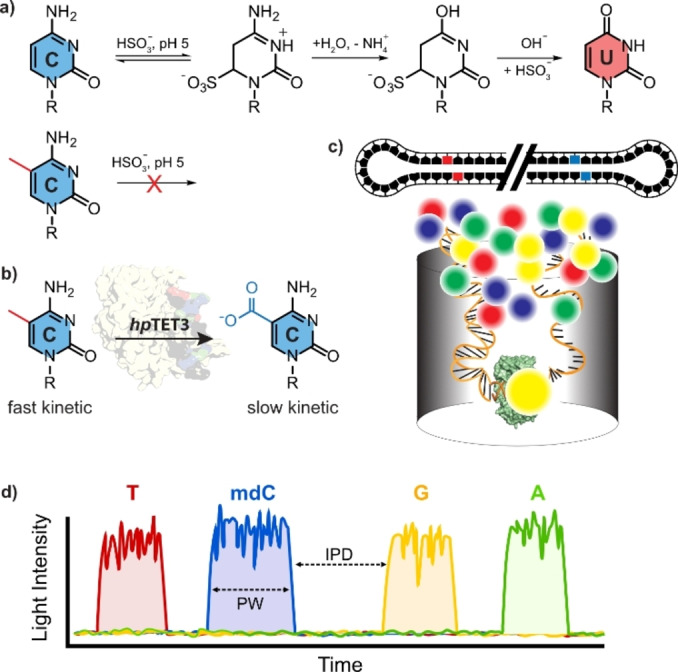
a) Depiction of bisulfite sequencing method and b) of the mdC to cadC oxidation chemistry as the basis for the sequencing method described in this publication. c) Depiction of the SMRT sequencing concept with a circular sequencing DNA template bound to the polymerase and fluorescent labeled triphosphates. d) Fluorescence signal during SMRT sequencing recorded by the Sequel IIe system. Y‐axis shows the fluorescent intensity, x‐axis the passing time. The kinetics or the nucleotide incorporation by the polymerase can be described by two time constants. The inter‐pulse‐duration (IPD), the time between two light pulses (nucleotide incorporation) and the pulse‐width (PW) the time the polymerase needs to form the phosphodiester bond. The presence of non‐canonical bases increase IPD and PW.

An alternative approach for sequencing mdC is third‐generation sequencing, where sequences are directly read without the need for a PCR step. Currently, all third‐generation single molecule sequencing tools, such as Nanopore or SMRT sequencing, allow direct read‐out of mdC.^[6]^

These methods, however, are still in their early stages, and the signal differences between the sequencing signals obtained for dC and mdC are often minimal. This necessitates cumbersome deconvolution of the data with substantial need for bioinformatics.[^7^, ^8^]

Thus, developing mild mdC sequencing methods that circumvent cytidine deamination is highly desirable for creating new early tumor diagnostic tools.

We hypothesized that the current limitations for mdC sequencing could be overcome by quantitatively oxidizing mdC to 5‐carboxycytidine (cadC, Figure 1b). Unlike dC, cadC has an additional carboxyl group, which is negatively charged under neutral pH‐conditions, supposedly providing a significant signal difference between the neutral mdC and the negatively charged cadC. The concept of detecting cadC instead of mdC by SMRT sequencing (Figure 1c) has been previously suggested^[9]^ but never implemented.

Here, we demonstrate that cadC significantly increases both kinetic parameters obtained during SMRT‐seq, inter‐pulse‐duration (IPD) and pulse‐width (PW), allowing us to train a deep‐learning algorithm. We found that the new method's accuracy surpasses all current mdC sequencing methods, paving the way for mild epigenetic mdC sequencing.

The foundation of this method is a newly developed truncated, yet robust Tet3 enzyme that can be overexpressed in *E. coli* and oxidizes mdC in the genome to cadC with over 99 % yield.

We implemented this new Tet‐based technology using SMRT sequencing, in which a polymerase pairs the nucleotides within the template with fluorescently labelled incoming triphosphates. A detector measures the fluorescent signal from the triphosphate bound in the polymerase's active site in real‐time before the fluorescence label is cleaved off during the phosphodiester bond formation process. Because the DNA fragment to be sequenced is embedded in a circular structure (Figure 1c), sequencing involves the polymerase moving along the circular template multiple times, so each base (including the cadC base) is read repeatedly, providing a large set of data points for each base and a high level of accuracy. Besides the fluorescence signal identifying the incoming base, SMRT‐sequencing also records the time the polymerase needs to form the phosphodiester bond (PW‐value) and the time between each incorporation event (IPD‐value), making multiple parameters available for each base to be sequenced, including cadC, which we form from mdC by Tet‐induced oxidation.

The first problem we addressed was developing a robust enzyme capable of oxidizing mdC quantitively to cadC. This was achieved using a ten‐eleven‐translocation enzyme, a Fe^2+^ and α‐ketoglutarate dependent monooxygenase.^[10]^ Until now, mdC to cadC oxidations were performed with difficult‐to‐overexpress Tet1 and Tet2 derived enzymes.[^5^, ^11^, ^12^, ^13^, ^14^] In contrast, in adult brains, the most prevalent Tet enzyme is Tet3,^[15]^ which oxidizes considerable amounts of mdC to 5‐hydroxymethyl‐dC (hmdC).^[16]^ We conducted a structure‐guided design based on the human Tet2 crystal structure^[17]^ (see SI) and identified a highly shortened (by ~72 %) mouse Tet3 variant (hpTet3) consisting of only 465 amino acids (52 kDa). In this hpTet3, we replaced the low‐complexity region within the catalytic domain (cd) with a glycine‐serine linker (Figure 2). The resulting hpTet3 protein could be overexpressed in *E. coli*. Similar approaches by two other groups[^18^, ^19^] notably deviate from our approach regarding the truncations made (see Supporting Information for further details). Fused to an *N*‐terminal Strep‐tag, we purified the hpTet3 first by affinity chromatography over StrepTrap XT material and subsequently used a Heparin column to remove chaperone contaminations (Hsp40 and Hsp70).

**Figure 2:**
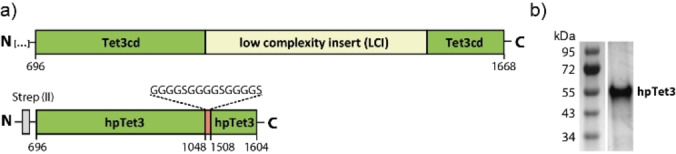
Purification of hpTet3. a) Depiction of the domain structure of hpTet3 in comparison to mouse Tet3. b) SDS‐PAGE of the purified protein after two steps (original image in Supporting Information Figure S2b).

Next, we investigated the catalytic performance of the hpTet3 enzyme. To this end, we first methylated lambda phage genomic DNA with the CpG‐specific methyltransferase M.SssI and digested it to the single nucleoside level. To quantify all nucleosides present in the genome, particularly potentially oxidized nucleosides such as 5‐formyl‐dC (fdC), 5‐hydroxymethyl‐dU (hmdU) and 8‐oxo‐dG, we performed quantitative triple quadrupole mass spectrometry (UHPLC‐QQQ‐MS) using a full set of internal, stable‐isotope labelled standards for dA, dT, dG, dC, mdC, hmdC, fdC, cadC, hmdU and 8‐oxo‐dG (Figure 3) using our previously described method.^[20]^ In a second experiment, we treated the genomic DNA with hpTet3 before digestion with hpTet3 (see Supporting Information for treatment conditions) and repeated the quantification experiment using the full set of isotope standards. This allowed us to obtain highly accurate quantitative data. As depicted in Figure 3a, we found that upon oxidation, the signal for mdC disappears, and a strong signal for cadC is detected. The intermediate oxidation states hmdC and fdC were not found, proving complete conversion. We repeated the study with various eukaryotic genomes (Figure 3b) and noted that in all cases the mdC (and 5‐hydroxymethyl‐dC) signal completely disappeared upon oxidation with hpTet3, generating a new and strong cadC signal.

**Figure 3:**
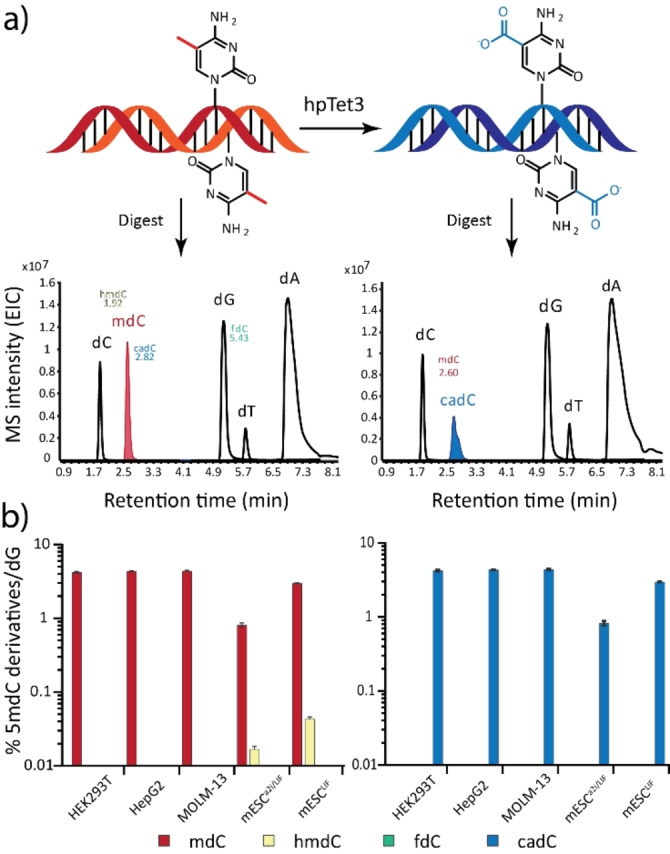
Oxidation of genomic DNA with hpTet3. a) M.SssI methylated λDNA(dam^‐^,dcm^‐^) was digested, isotope standards were added (see Supporting Information for complete list) and the mixture analyzed by isotope dilution triple quadrupole mass spectrometry giving quantitative data for all nucleosides (left panel). The quantitative analysis of the nucleoside composition of genomic DNA was repeated after treatment of the genomic DNA with hpTet3 (right panel). b) This analysis was performed on various human and mouse genomes with natural methylation levels. Depicted are mean values ±s.d. of biological replicates (*n*=3). Bars represent the mean and error bars show the standard deviation.

In all cases, we were unable to detect residual mdC. Instead, cadC was detected at levels mirroring those of mdC found in the input material e.g. 4.23 % in HEK293T gDNA (see Supporting Information Table S1 for other samples). Quantitative analysis of the hpTet3 reaction revealed an mdC to cadC oxidation yield of 99.96 % within genomic DNA.

To estimate unwanted oxidative damage, we quantified the level of 8‐oxo‐dG and observed no significant increase. For hmdU, we observed an expectedly increase to 48 hmdU DNA lesions per genome.^[21]^ These data demonstrate that the mdC to cadC oxidation with hpTet3 is highly efficient, with a small number of generated hmdU lesions (SI Figure S3 & Table S2).

With this result, we next explored the possibility to sequence mdC using third‐generation SMRT sequencing. For SMRT sequencing, we prepared three model genomes from lambda phage DNA (dam^‐^, dcm^‐^). The first genome (LMD‐dC) contained no mdC. In the second genome, we enzymatically methylated all CpG sites with M.SssI (LMD‐mdC). For the third genome, we oxidized the LMD‐mdC genome with hpTet3 to convert all mdCs to cadCs (LMD‐cadC). To monitor the purity of these model DNAs, we digested the three genomes to the nucleoside level and analyzed the nucleoside composition using UHPLC‐QQQ‐MS. The obtained data (SI Table S1) confirmed the high methylation efficiency of M.SssI (99.99 %) and the high oxidation efficacy to cadC using hpTet3 (99.96 %).

Following library preparation and sequencing on a Sequel IIe platform, we obtained 385,942 reads for the unmodified lambda genome, 433,815 reads for the mdC‐containing genome and 456,646 reads for the cadC containing genome. Next, we performed alignment feature extraction (IPD & PW values) using ccsmeth (https://github.com/PengNi/ccsmeth) as shown in Figure 4a. We extracted mean IPD and PW values for each CpG position in the lambda genome within a 21‐*k*‐mer (±10 bp around the cytosine of interest) (Figure 4b). Regarding the IPD values, we observed a large difference in the 21‐*k*‐mer between the dC, mdC and cadC situations (Figure 4b left). Compared to dC, mdC and cadC showed different signal patterns near the xdC position (*k*‐mer‐positions 8‐19). While the signal patterns for mdC and cadC are similar, the normalized time values are strongly increased for cadC. Additionally, we observed differences in the PW patterns (Figure 4b right). Notably, cadC shows a strong time increase at the *k*‐mer‐position 18, which is 7 positions downstream from the cadC position. This data shows how complex the footprint differences are between the dC, mdC and cadC situations, particularly outside of CpG dyads. The PW and IPD difference are manifested not only at the nucleotide itself but also several nucleotides away, up‐ or downstream (SI Figure S4). To ensure these patterns are not the result of overlapping signals from neighboring CpG sites, we plotted IPD and PW for 21‐*k*‐mers containing one, two or more than two CpGs (SI Figure S5). In all cases, the extracted patterns for IPD and PW are similar, indicating multiple and complex interactions between the polymerase and the template.

Next, we explored whether the complex, but strong kinetic PW und IPD data for cadC could be used to train an AI‐based recurrent neural network (RNN). Following the training pipeline of ccsmeth, we trained a mdC‐model based on the LMD‐dC and LMD‐mdC datasets, as well as a cadC‐model based on the LMD‐dC and LMD‐cadC datasets (Figure 4a). The key parameter obtained from the AI‐model are shown in Supporting Information Figure S6.

To our delight, we discovered that the cadC kinetic sequencing data in combination with the trained algorithm provided a cadC‐model that exceeds the performance of the canonical ccsmeth and our mdC‐LMD model in all aspects. To directly detect mdC, the RNN required 181 training rounds to reach a model accuracy of 0.945. The precision reached a value of 0.962 and the recall (number of describable CpG dyads) was 0.956. For cadC, the model needed only 16 training steps to obtain an accuracy of 0.987, a precision of 0.987, and a recall of 0.988. These are fantastic values particularly in light of the few required training steps (SI Figure S6).

We next tested the new models by applying them to the individual datasets obtained for LMD‐dC, LMD‐mdC and LMD‐cadC. For the detection of mdC, we compared our LMD‐mdC and LMD‐cadC models with the standard ccsmeth‐model, which is trained on human DNA^[22]^ Based on our UHPLC‐QQQ‐MS measurements, we know that the modified genetic material underlying the LMD‐mdC and LMD‐cadC models contain 99.99 % mdC and 99.96 % cadC in the CpG dyads, respectively (SI Table S1). In the LMD‐mdC model, the methylation frequency of a CpG dyad should consequently be almost 1, while it should be close to 0 in the non‐methylated LMD‐dC DNA. The sequencing results are summarized in Figures 4c and S7 with details shown in Supporting Information Tables S3 and S4.

**Figure 4:**
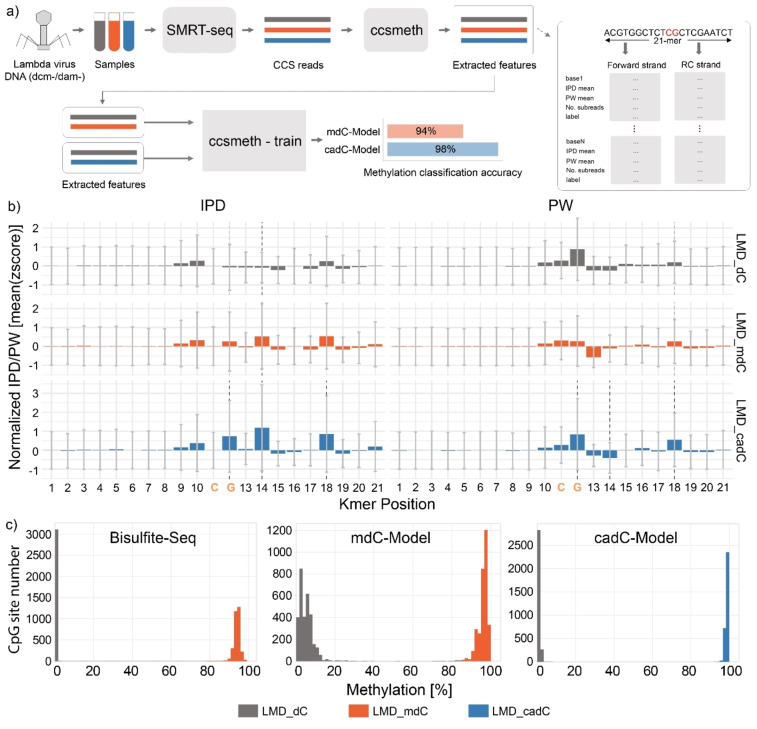
Application of hpTet3 for SMRT sequencing. a) sequencing and model training workflow; dcm & dam negative DNA from lambda phage were sequenced using the Sequel IIe system, HiFi reads were aligned and IPD as well as PW values (features) extracted using ccsmeth; With ccsmeth, we trained a 5mdC detecting model, as well as a 5cadC detecting model. b) mean IPD & PW values (zscore normalized) extracted by ccsmeth across a 21‐*k*‐mer centered around a given CpG for unmodified λDNA (LMD‐dC, grey), methylated λDNA (LMD‐mdC, red) and carboxylated λDNA (LMD‐cadC, blue). c) methylation frequency of the λ genome using three analysis methods. The left graph shows Bisulfite‐Seq data for LMD‐mdC and LMD‐dC. The middle graph shows the methylation frequency (Pacbio's CCS) of LMD‐mdC and LMD‐dC using a custom‐trained mdC‐model. The right graph represents the methylation frequency (Pacbio's CCS) of M.Sssl methylated, hpTet3‐oxidized λDNA (LMD‐cadC) and LMD‐dC with a custom‐trained cadC‐model.

As a reference, we sequenced LMD‐dC and LMD‐mdC with bisulfite to compare our results obtained by the individual ccsmeth pipeline. The bisulfite data indicate that 98.2 % of all CpGs show a methylation frequency of >=90 %. However, only 23.82 % of all CpGs show a methylation frequency of >=95 %.

Analyzing the SMRT‐seq data with our mdC model, we found that 76.75 % of CpG sites in the LMD‐mdC sample were reported to have a methylation frequency >95 %. Additionally, 21.1 % of the CpGs in the LMD‐dC sample were reported to have >10 % methylation. The numbers were even lower when we applied the canonical ccsmeth model, where only 30 % of the CpG sites were reported with a methylation frequency of >95 % (SI Table S3).

With the new cadC‐model trained on hpTet3‐oxidized lambda DNA, we detect a sharp signal (blue) of nearly 100 % for the cadC frequency in CpG dyads (Figure 4c). Specifically, 94.68 % of all CpGs from the LMD‐dC sample have a modification frequency of <10 % and 99.95 % of all CpGs from the LMD‐cadC sample have a modification frequency of >=95. In fact, our model predicts that 94.63 % of all CpGs have a methylation frequency of >=98 %.

These results show that the new model is able to predict all the cadCs in the lambda genome with unprecedented accuracy, which is based on the fact that the oxidation of mdC to cadC with the new hpTet3 enzyme provides highly characteristic PW and IPD values. This, in combination with the AI‐derived cadC‐model enables highly accurate and sensitive sequencing of mdC. In contrast to other bisulfite‐free methods,[^5^, ^11^, ^23^] our approach is one‐step and PCR free, reducing potential bias.

We found that the sequence‐dependent oxidation bias introduced by the hpTet3 enzyme (SI Figs. S8 & S9 and Supporting Information Table S5 & S6) is small, especially when high enzyme concentrations are used. This is another reason for the high accuracy (99,9 %) of our 5mdC sequencing method.

In summary, we describe the development of a new hpTet3 variant, that can be overexpressed in *E. coli*. hpTet3, is stable and oxidizes mdC to cadC with high efficiency. The sequence bias for the oxidation is low. Especially with higher enzyme concentrations, we are able to oxidize mdCs to cadC with high efficiency, even in non‐CpG contexts. Thus, in principle, the enzyme can support mdC‐sequencing in non‐CpG contexts in combination with a deamination reaction and PCR. We performed SMRT sequencing of hpTet3 treated DNA and saw highly characteristic IPD and PW values that allowed the precise localization of cadC in all CpG contexts. SMRT sequencing of mdC via cadC was achieved with unprecedented accuracy after further manipulation. Another strength of the method is the ability to perform long‐read sequencing, which gives highly accurate data also for repetitive genome elements. The new hpTet3 can in addition help to improve other 5mdC sequencing strategies, such as TAPS and EM‐Seq. By analogy, we termed our method Enzymatic Methyl oxidation sequencing (EMox‐seq).

## Conflict of Interests

The authors declare no conflict of interest.

## Supporting information

As a service to our authors and readers, this journal provides supporting information supplied by the authors. Such materials are peer reviewed and may be re‐organized for online delivery, but are not copy‐edited or typeset. Technical support issues arising from supporting information (other than missing files) should be addressed to the authors.

Supporting Information

## Data Availability

The data discussed in this publication is provided in the Supporting Information. Sequencing data have been deposited in NCBI′s Gene Expression Omnibus^[24]^ and are accessible through GEO Series accession number GSE256446 (https://www.ncbi.nlm.nih.gov/geo/query/acc.cgi?acc=GSE256446).
