# Supplementary material for: Robust Bisulfite‐Free Single‐Molecule Real‐Time Sequencing of Methyldeoxycytidine Based on a Novel hpTet3 Enzyme
Source: Angew Chem Int Ed Engl. 2024 Nov 25;63(52):e202418500. doi: 10.1002/anie.202418500 (PMC11656142; doi:10.1002/anie.202418500)
Supplement: Supplementary file 1 — Supporting Information [file ANIE-63-e202418500-s001.pdf]

Angewandte Chemie
Eine Zeitschrift der Gesellschaft Deutscher Chemiker

Supporting Information

Robust Bisulfite-Free Single-Molecule Real-Time Sequencing of Methyldeoxycytidine Based on a Novel hpTet3 Enzyme

H. Sahin, R. Salehi, S. Islam, M. Müller, P. Giehr, T. Carell**

SUPPORTING INFORMATION

TABLE OF CONTENTS

Supporting Information 1
Supplementary Methods 2
Structure-guided design of hpTet3 2
Expression and purification of hpTet3 in *E.coli* 2
Preparation of methylated lambda DNA 3
hpTet3 oxidation 4
Cell culture 5
Isolation of gDNA 5
DNA digestion 5
UHPLC-QQQ-MS 5
SMRTseq - Sequel IIe sequencing 6
Whole-genome Bisulfite Sequencing 8
Supplementary Figures 10
Supplementary Tables 16
Supplementary References 19

Supplementary Methods

Structure-guided design of hpTet3

All three TET proteins share a common domain architecture consisting of a large and mostly uncharacterized N-terminal part and a highly conserved C-terminal catalytic domain (cd) composed of a Cys-rich region and a double-stranded β helix domain (DSBH). The DSBH domain is divided into two parts, separated by a large and presumably unstructured low-complexity insert (LCI) of unknown function and is preceded by the cysteine-rich region. Hu *et al*.[1] solved the crystal structure of a truncated, but catalytically active form of human Tet2 (hTet2) in complex with a 5mC-DNA duplex and identified the minimal regions which are required for the catalysis of human Tet2 by measuring the catalytic activity of various N-terminal, C-terminal truncations, and deletions of the low-complexity insert. In an attempt to map the minimal catalytically active fragment of mouse Tet3, various bioinformatic tools for aligning protein sequences, predicting secondary structures and disordered regions were used and results were compared to the hTet2 crystal structure. To avoid intrinsically disordered or unstructured protein regions within the Tet3 sequence and thereby to increase solubility, stability and improve folding during protein expression, these fragments were predicted using the DisEMBL computational tool.[2] PSIPRED[3] was used to predict secondary structures. The minimum regions necessary for catalysis for mouse Tet3 we determined are 696 – 1604, Δ1048 - 1508.

Notably, two other groups designed truncated mTet3 variants. Figure S1 shows a sequence alignment of Tet3cd (including the LCI), hpTet3 and truncated Tet3 from M. Ravichandran *et al*.[4] and B. Sudhamalla *et al*.[5] We deviated from M. Ravichandran *et al*. primarily in terms of the internal deletion of the LCI and in the C-terminus. The authors deleted 207 amino acids (aa) in the LCI, whereas a deletion of 459 aa was made by us. Additionally, no truncations of the C-terminus were made by M. Ravichandran *et al*. In contrast, in hpTet3 the C-terminus was deleted by 64 aa. Their construct contained the following aa: 693-1668 Δ 1302-1509. hpTet3 differed from B. Sudhamalla Tet3 particularly in the truncation made in the LCI and minor differences in the N- and C-terminal part.

Expression and purification of hpTet3 in *E.coli*

A plasmid encoding an N-terminally Strep(II)-tagged truncated mouse Tet3 protein (hpTet3: 696 – 1604 with residues 1048 – 1508 replaced by a 15-residue GS-linker GGGGSGGGGSGGGGS) was designed and sub-cloned into pET28a between the NcoI and XhoI restriction sites. The hpTet3 sequence was codon optimized for expression in *E.coli* and purchased from Life Technologies. In preparation of recombinant protein expression, BL21 (DE3) competent *E. coli* cells were transformed with Strep(II)-tagged hpTet3 plasmid and selected on Luria-Bertani (LB) plates containing kanamycin (25 µg/ml final concentration). A single colony was picked and cultured overnight at 37 °C in 50 mL of LB broth with appropriate antibiotic at 37 °C at 180 rpm in an Innova S44i incubator shaker (Eppendorf). For protein expression, bacteria from the overnight small-scale culture were diluted 1000-fold in LB medium with appropriate antibiotic and cells were grown at 37°C and 200 rpm until an optical density (OD_{600}) of 0.3 was reached. Then cultures were cooled down to 16°C and target protein expression was induced at an OD_{600} between 0.5 and 0.6 by addition of isopropyl-b-D-thiogalactopyranoside to a final concentration of 0.5 mM. Furthermore, cells were supplemented with a 1000x trace element stock solution to a concentration of 1x trace metals containing 50 µM $FeCl_2$, 10 µM $ZnSO_4$, 20 µM $CaCl_2$, 10 µM $MnCl_2$, 2 µM $CoCl_2$, 2 µM $CuCl_2$, 2 µM $NiCl_2$, 2 µM Na_2MoO_4, 2 µM Na_2SeO_3 and 2 µM H_3BO_3. Protein expression was carried out for 18 h. Subsequently, cells were harvested by centrifugation at 8000 rpm for 10 min at 4 °C, rinsed in cold PBS, then pelleted again at 8000 rpm. The pellet was flash-frozen in liquid nitrogen and stored at -80 °C until further use.

For purification, frozen cell pellets were thawed on ice, resuspended in ice-cold lysis buffer (50 mM HEPES pH 6.8, 500 mM NaCl, 10% glycerol, 0.5 mM TCEP, 10 µM $ZnCl_2$, 0.5 mg/ml lysozyme,

EDTA-free protease inhibitor cocktail (Roche), 5 mM ATP and 5 mM $MgCl_2$) and incubated on ice for 30 min. To reduce viscosity from chromosomal DNA and RNA, lysates were treated with Benzonase. Cells were lysed using a high-pressure homogenizer (EmulsiFlex C5) and the cell lysate was cleared by centrifugation at 40,000 g for 45 min at 4 °C. Cleared lysate was loaded on a StrepTrap XT column (Cytiva 5 mL column) for affinity purification. After sample application, the column was washed with 8 column volumes (CV) of wash buffer (50 mM HEPES pH 6.8, 500 mM NaCl, 10% glycerol and 0.5 mM TCEP) and bound protein was eluted with 10 CV buffer containing 50 mM HEPES (pH 6.8), 100 mM NaCl, 10% glycerol, 0.5 mM TCEP and 50 mM biotin. Fractions containing hpTet3 were pooled and concentrated with an Amicon ultra centrifugal filter device (30 000 MWCO, 15 mL). Then, the eluate was diluted 1-fold with a buffer containing 50 mM HEPES pH 6.8, 100 mM NaCl, 10% glycerol, 10 mM ATP, 10 mM $MgCl_2$ and 0.5 mM TCEP and incubated for 30 min on ice. This procedure aims at the removal of contaminating tightly bound molecular chaperone Hsp70 (DnaK) – Hsp40 (DnaJ) – hpTet3 complexes. Subsequently, incorrectly folded hpTet3 aggregates were removed by centrifugation for 30 min at 25,000 g at 4°C and the supernatant was applied to a HiTrap Heparin HP column (Cytiva, 5 ml) pre-equilibrated in binding buffer (50 mM HEPES pH 6.8, 100 mM NaCl, 10% glycerol (v/v), 0.5 mM TCEP). The column was washed with 20 CV of binding buffer and hpTet3 was eluted with a linear salt gradient ranging from 0.1 to 1.5 M NaCl (0%–100% elution buffer containing 50 mM HEPES pH 6.8, 1.5 M NaCl, 10% glycerol (v/v), 0.5 mM TCEP) over 20 CV. hpTet3-containing fractions were buffer exchanged and concentrated to a final buffer containing 50 mM HEPES pH 6.8, 250 mM NaCl, 10% glycerol and 0.5 mM TCEP using Amicon centrifugal filters with a molecular weight cut-off of 30 kDa (EMD Millipore). Aliquots of pure hpTet3 were flash-frozen in liquid nitrogen and stored at -80 °C. Protein purity was confirmed by Coomassie-stained SDS-PAGE. Typical yields were 2-3 mg per liter of *E. coli* culture.

All column chromatography steps were carried out at 4 °C with pre-cooled buffers on a ÄKTA pure chromatography system and protein-containing fractions were kept on ice during whole purification procedure.

Preparation of methylated lambda DNA

For Sequel IIe Sequencing

Lambda DNA (Oxford Nanopore: EXP-CTL001) was amplified by whole genome amplification (WGA) using the Direct WGA kit (Jena Bioscience, cat. no. PCR-382S) in order to produce unmodified DNA. The WGA was conducted according to Direct WGA kit's protocol - 1.0 ng lambda DNA was incubated with 12 µl reaction buffer, 1 mM dNTP mix, 1 µl primer mix and 1 µl enzyme mix in a 20 µL reaction volume at 30°C for overnight (16 h) and followed by a heat inactivation for 5 min at 65°C.

Unmodified lambda DNA was sheared by Bioruptor® Pico (Diagenode) (2 µg lambda DNA, 2 times of 2 cycles at 5”/90”) to produce 1 kb size of fragmented DNA.

1 µg of unmethylated, sheared DNA was methylated in vitro using 4 U of M.SssI enzyme (NEB, cat. no. M0226S) in the presence of 160 µM S-adenosylmethionine (SAM) (NEB, cat. no. B9003S) in the provided reaction buffer (1x) (10 mM Tris-HCl pH 7.9, 50 mM NaCl, 10 mM $MgCl_2$ and 1 mM DTT) (NEB, cat. no. B7002S) in a 50 µL reaction volume for 90 min at 37°C. To ensure complete CpG methylation, the reaction mixture was supplemented with additional 4 U of M.SssI, 145 µM SAM and the reaction buffer (0.1x) in a 55 µL reaction volume. Then the reaction was incubated for an additional 90 min at 37°C followed by a heat inactivation for 20 min at 65°C. Methylated DNA was purified with 1.8x AMPure XP beads (Beckman Coulter, pro. no. A63882) according to the manufacturer's protocol. DNA methylation was confirmed by quantitative UHPLC-QQQ-MS/MS.

To assess the sequence preference of hpTet3 in the oxidation of non-CpG sites

Lambda DNA (1 µg) was methylated *in vitro* using the following methyltransferases according to the manufacturer's recommended protocol in a 50 µL reaction volume for 2 h at 37°C using 5 U of the respective methyltransferase enzyme:

AluI (NEB, cat. no. M0220S) methylates cytosines in a 5' - AG**C**T - 3' context.

HaeIII (NEB, cat. no. M0224S) methylates internal cytosines of the 5' -GG**C**C- 3' sequence context.

MspI (NEB, cat. no. M0215S) methylates external cytosines in a 5'… **C**CGG …3' sequence context.

The GpC methyltransferase M.CviPI (NEB, cat. no. M0227S) methylates all cytosines in a 5'…GpC…3'dinucleotide sequence.

Subsequently, after 2 hours of initial incubation to ensure complete methylation of all potential sites, the reaction mixture was supplemented with 5 U of the respective methyltransferase, 145 µM SAM and the reaction buffer (0.1x) in a 55 µL reaction volume and incubated for additional 2 hours. Methylated DNA was purified with 1.8x AMPure XP beads according to the manufacturer's protocol. Methylation efficiency was determined by quantitative UHPLC-QQQ-MS/MS as described for lambda DNA methylated using the methyltransferase M.SssI.

For Whole genome Bisulfite Sequencing (WGBS)

Unmodified lambda DNA (Thermo Fisher, cat. no. SD0021), methylated lambda DNA in CpG context (catalyzed by methyltransferase M.SssI) and methylated lambda in non-CpG context (catalyzed by methyltransferase M.CviPI) were sheared by Bioruptor®Pico (Diagenode) (1 µg lambda DNA, 2 times of 2 cycles at 15"/90") to produce ~ 300 bp size of fragmented DNA. Quantitative UHPLC-QQQ-MS/MS confirmed DNA methylation.

hpTet3 oxidation

All reactions were performed with 1 µg genomic DNA in a total volume of 50 µl at 37 °C for 1h at 500 rpm in a thermomixer. DNA concentrations were quantified using 1x dsDNA-HS (High Sensitivity) Qubit Assay-Kit.

1 µg of human or mouse genomic DNA was incubated with 4 µM (10 µg) recombinant hpTet3 protein in buffer containing 50 mM HEPES, 50 mM NaCl, 1 mM α-ketoglutarate, 2 mM ascorbic acid, 1.2 mM ATP, 105 µM $Fe(NH_4)_2(SO_4)_2$ and 2.5 mM DTT at 37°C for 1 h. The pH of the reaction was 7.4 after addition of all buffer components. Alternatively, since 25.7% of total cytosines are methylated (mdC) in M.SssI treated lambda DNA, compared to approximately 4.5% of mdC in human gDNA, 1 µg of M.SssI treated lambda DNA was incubated with 11 µM (30 µg) recombinant hpTet3 at 37°C for 1 h with the same buffer condition. After that, 0.8 U of Proteinase K (NEB, cat. no. P8107S) and SDS to a final concentration of 0.05% were added to the reaction mixture and incubated for 1h at 50 °C. Subsequently, oxidized DNA was purified with 1.8x AMPure XP beads (Beckman Coulter) according to the manufacturer's protocol. Oxidation efficiency was quantified by UHPLC-QQQ-MS/MS.

hpTet3 oxidations to assess the catalytic activity of hpTet3 in a non-CpG sequence context were performed under saturated conditions using 1 µg of *in vitro* methylated lambda DNA (see above) and 15 µM (40 µg) recombinant hpTet3 in the same buffer as specified above at 37°C for 90 min. The pH of the reaction was 7.2 after adding all buffer components.

It is important to note that ascorbic acid and $Fe(NH_4)_2(SO_4)_2$ must be freshly prepared and Fe(II) should be dissolved in water and added immediately before the reaction begins to minimize the oxidation to Fe(III).

For Whole-genome Bisulfite Sequencing (WGBS)

hpTet3 oxidized lambda DNA samples, in both CpG and non-CpG contexts, were also sheared by Bioruptor® Pico (Diagenode) (1 µg lambda DNA, 2 times of 2 cycles at 15"/90") to produce ~ 300 bp size of fragmented DNA. Quantitative UHPLC-QQQ-MS/MS confirmed DNA oxidation.

Cell culture

All cell lines used were cultivated at 37 °C in water saturated, CO_2-enriched (5%) atmosphere.

HEK293T cells (CLS) and human hepatocellular carcinoma HepG2 cells (CLS) were grown in DMEM with high glucose content (Sigma-Aldrich D6546), supplemented with 10% (v/v) fetal bovine serum (FBS) (Life Technologies 10500-064), 1% (v/v) L-alanyl-L-glutamine (Sigma-Aldrich G8541), and 1% (v/v) penicillin–streptomycin (Sigma-Aldrich P0781). Cells were passaged twice a week at a ratio of 1:10 when reaching a confluence of 70–80%.

MOLM-13 cells were grown in RPMI 1640 (Sigma-Aldrich R0883), containing 10% (v/v) FBS (Invitrogen 10500–064) and 1% (v/v) L-alanyl-L-glutamine (Sigma-Aldrich G8541). The cells were routinely passaged in a ratio of 1:6 to 1:10 when a density of 2×10^6 cells/mL was reached.

J1 mESCs were cultivated on 0.2% (w/v) gelatine-coated plates in DMEM (Sigma-Aldrich D6546), supplemented with 10% (v/v) Pansera ES-grade FBS (Pan Biotech), 1× MEM-nonessential amino acids (NEAA, Sigma-Aldrich M71145), 2 mM L-alanyl-L-glutamine, 1x Penicillin-Streptomycin (Sigma-Aldrich AP078), 0.1 mM β-mercaptoethanol, 10^3 U/mL mouse recombinant LIF (mLIF, Sigma-Aldrich ESG1107), 1.5 µM CGP 77675 (Sigma-Aldrich SML0314) and 3 µM CHIR 99021 (Axon Medchem) (a2iL conditions). mESCs were maintained in the naïve state in a2iL medium and passaged every 2 – 3 d in a ratio of 1:4 to 1:8 when a confluency of 60 – 75% was reached. To shift cells from the hypomethylated naïve state to a primed state with increased mdC and hmdC levels, cells were cultured in medium supplemented with FBS and LIF as described above but in the absence of GSK3α/β and Src kinase inhibitor. Cells were primed for 72 h in total before gDNA isolation.

Cells were tested for Mycoplasma contamination at least every 2 months.

Isolation of gDNA

Cells were lysed directly in the plates with RLT buffer (Qiagen) supplemented with 0.01 equiv. of 2-Mercaptoethanol (14.3 mM final concentration), antioxidants 3,5-di-tert-butyl-4-hydroxytoluene (BHT, 200 µM) and deferoxamine mesylate salt (Desferal, 200 µM). To further homogenize the lysate and shear the gDNA, samples were subjected to bead milling using a Qiagen TissueLyser for 30s at 30 Hz. After cell lysis, gDNA isolation was performed as previously described in Traube *et al.*[6] Isolated gDNA was subjected to nucleoside digest and UHPLC-QQQ-MS/MS measurement before and after hpTet3 oxidation.

DNA digestion

The purified DNA products were digested to nucleosides with the Nucleoside Digestion Mix from NEB (M0649S) in a total volume of 50 µL using 1 µL of enzyme and 5 µL of 10x reaction buffer at 37°C for 2h. Samples were filtered by using an AcroPrep Advance 96-well Supor filter plate, 0.2 µm (Pall Life Sciences) and subjected to UHPLC-QQQ-MS/MS.

UHPLC-QQQ-MS

Absolute quantification of modified nucleosides was performed with a previously published method.[6] For the exact quantification of nucleosides using the stable isotope dilution technique, an Agilent 1290 Infinity II equipped with a variable wavelength detector (VWD) combined with an Agilent Technologies G6490 Triple Quad LC/MS system with electrospray ionization (ESI-MS, Agilent Jetstream) was used. Chromatography was performed using a Poroshell 120 SB-C18 column (2.7 µm, 2.1 × 150 mm; Agilent

Technologies, cat. no. 683775-902) at 35 °C and a flowrate of 0.35 mL/ min using water supplemented with 0.0075% (vol/vol) formic acid (FA) as solvent A and acetonitrile (MeCN) supplemented with 0.0075% (vol/vol) FA as solvent B. The gradient started at 100 % solvent A, followed by an increase to 3.5% solvent B over 4 min (0 min – 4 min). From 4 min to 7 min solvent B was increased to 5% and from 7.0 min to 7.5 min, solvent B was increased further to 80 %, maintained at 80 % for 2.0 min before returning to 100 % solvent A in 0.5 min and a 3.0 min re-equilibration period. The operating parameters were: positive-ion mode, cell accelerator voltage of 5 V, N_2 gas temperature of 120 °C, N_2 gas flow of 11 L/min, sheath gas temperature of 280 °C with a flow of 11 L/min, capillary voltage of 3000 V, nozzle voltage set to 0 V, nebulizer at 60 psi, high-pressure RF at 150 V and low-pressure RF at 60 V. The instrument was operated in dynamic MRM mode. The fragmentor voltage was 380V for all compounds, while other compound-dependent parameters are summarized in Supplementary Table S1 together with the retention times and mass transitions of unlabeled and isotope-labeled nucleosides. MS1 resolution was set to "Wide" and the MS2 resolution to "Unit. Each sample was co-injected with 1 µL of 0.5 µM stable isotope-labeled internal standard (ISTD) mix containing the following isotope standards: [$^{15}N_5$-$^{13}C_{10}$]-dA, [$^{13}C_9$]-dC, [$^{15}N_5$-$^{13}C_{10}$]-dG, [$^{15}N_2$-$^{13}C_{10}$]-dT, [D_3]-m^{5}dC, [D_2-$^{15}N_2$]-hm^{5}dC, [$^{15}N_2$]-f^{5}dC, [$^{15}N_2$]-ca^{5}dC, [$^{15}N_5$]-8-oxo-dG and [D_2]-hmdU. The sample data were analyzed by Agilent's Quantitative MassHunter Software (v B07.01) using the built-in calibration function.

Compound-dependent LC-MS/MS-parameters. R_t: retention time CE: collision energy; CAV: collision cell accelerator voltage.

Compound	Precursor ion (m/z)	MS1 resolution	Product ion (m/z)	MS2 resolution	R_t (min)	CE (V)	CAV (V)	Polarity
**[$^{15}N_2$]-cadC**	274.08	Wide	158.03	Unit	2.9	6	5	Positive
**cadC**	272.09	Wide	156.04	Unit	2.9	6	5	Positive
**[D_2-$^{15}N_2$]-hmdC**	262.12	Wide	146.07	Unit	1.9	4	5	Positive
**hmdC**	258.11	Wide	142.06	Unit	1.9	4	5	Positive
**[D_3]-mdC**	245.13	Wide	129.09	Unit	2.6	4	5	Positive
**mdC**	242.11	Wide	126.07	Unit	2.6	4	5	Positive
**[D_2]-hmdU**	261.08	Wide	145.1	Unit	3.7	4	5	Positive
**hmdU**	259.08	Wide	143.1	Unit	3.7	4	5	Positive
**[$^{15}N_5$]-8oxodG**	289.09	Wide	173.04	Unit	6.1	9	5	Positive
**8oxodG**	284.1	Wide	168.05	Unit	6.1	9	5	Positive
**[$^{15}N_2$]-fdC**	258.09	Wide	142.04	Unit	5.4	5	5	Positive
**fdC**	256.09	Wide	140.05	Unit	5.4	5	5	Positive
**dC**	228.1	Wide	112.1	Unit	1.7	5	5	Positive
**[$^{13}C_9$]-dC**	237.1	Wide	116.1	Unit	1.7	5	5	Positive
**dA**	252.1	Wide	136.1	Unit	6.9	12	5	Positive
**[$^{15}N_5$-$^{13}C_{10}$]-dA**	267.1	Wide	146.1	Unit	6.9	12	5	Positive
**dG**	268.0	Wide	152.1	Unit	5.1	6	5	Positive
**[$^{15}N_5$- $^{13}C_{10}$]-dG**	283.1	Wide	162.06	Unit	5.1	6	5	Positive
**dT**	243.1	Wide	127.1	Unit	5.7	3	5	Positive
**[$^{15}N_2$- $^{13}C_{10}$]-dT**	255.1	Wide	134.1	Unit	5.7	3	5	Positive

SMRTseq - Sequel IIe sequencing

Libraries for the Sequel IIe were prepared according to PacBio's SMRTbell express template prep kit 2.0 (PN: 100-938-900). Briefly, for DNA-repair and A-tailing, 300 ng - 1 µg DNA (1 kb) in 46 µl nuclease free water, 8 µl repair buffer, 4 µl end repair mix and 2 µl DNA repair mix were mixed by pipetting and

incubated at 30°C for 30 min. The reaction was inactivated by incubating at 65°C for 5min. Next, 4 µl SMRTbell barcoded adapter (Barcoded overhang adapter kit 8B, PN: 101-628-500), 30 µl ligation mix and 1 µl ligation enhancer were mixed by pipetting. 31µl of the ligation mix was directly added to the A-tailed DNA. The reaction was incubated for 30 min at 20°C and subsequently purified using SMRTbell cleanup beads. Non-SMRTbell-DNA was depleted by nuclease treatment. The concentration of the final library was determined using the Qubit dsDNA-HS-Assay-Kit (ThermoFisher: cat. no. Q32851). Sequencing was conducted on a Sequel IIe platform from PacBio.

Subreads obtained from Sequel IIe sequencing were processed using ccsmeth (https://github.com/PengNi/ccsmeth).[7] First, subreads were converted to ccs reads containing kinetics (IPD and PW values) using the "ccsmeth call_hifi" function and mapped to the lambda genome using "ccsmeth align_hifi" function. For model training, feature tables were produced using "ccsmeth extract". Individual models were trained applying the "ccsmeth train" function on features from either LMD-C and LMD-mC (mdC-Model) or LMD-C and LMD-cadC (cadC-Model). Random initialization and training on 75% of the datasets were conducted, with 75% of each of samples used for optimizing parameters until loss reached a minimum for inference. Subsequently, performance was assessed on the remaining 25% (testing set). We used a learning rate of $1x10^{-3}$ and stopped training after no improvement in test loss could be seen for 4 epochs, allowing a maximum of 20 epochs. The individual models were applied using the "ccsmeth call_mods" and "ccsmeth call_freqb" functions. Model parameter and methylation frequency was plotted using R. Below, we provide the ccsmeth code for processing the LMD_dC reads. LMD_mdC and LMD_caC reads were processed the same way.

- Hifi read calling with kinetics from subreads.

```
ccsmeth call_hifi –subreads ./LMD_dc_subreads.bam \
 --threads 10 \
 --output ./LMD_dc.hifi.bam
```

- Hifi read alignment.

```
ccsmeth align_hifi --hifireads ./LMD_dc.hifi.bam \
 --ref ./GCF_000840245.1_ViralProj14204_genomic.fa \
 --output ./LMD_dc.hifi.pbmm2.bam \
 --threads 10
```

- Feature extraction.

```
ccsmeth extract --input ./LMD_dc.hifi.pbmm2.bam \
 --ref ./GCF_000840245.1_ViralProj14204_genomic.fa \
 --output ./LMD_dc_features.tsv \
 --norm zscore \
 --methy_label 0\
 --mode align \
 --motifs CG \
 --threads 10
```

When the model is trained, the following step is providing call modification:

- Modification calling. You need to run the code on GPU,

```
ccsmeth call_mods --input ./LMD_dc.hifi.pbmm2.bam \
 --ref ./GCF_000840245.1_ViralProj14204_genomic.fa \
 --output ./LMD_dc_.hifi.pbmm2.call_mods \
 --threads_call 2 \
 --model_file ./LMD_dc_ccsmeth.ckpt\
 --mode align \
```

```
--model_type attbigru2s \
--threads 10
```

- Methylation frequency calling

```
ccsmeth call_feqb --input ./LMD_dc.hifi.pbmm2.call_mods.modbam.bam \
 --ref ./GCF_000840245.1_ViralProj14204_genomic.fa \
 --output ./LMD_dc_.hifi.pbmm2.call_mods.modbam.freq \
 --sort --bed --call_mode aggregate \
 --aggre_model ./model_aggregate.ckpt \
 --threads 10
```

Whole-genome Bisulfite Sequencing

The Whole-genome Bisulfite Sequencing (WGBS) libraries were prepared as follows. For A-tailing, 200 ng sheared DNA (~ 300 bp) in 25 µl ddH_2O (MilliQ), 3.5 µl NEBNext Ultra II End Prep Reaction Buffer (NEB, cat. no. E7546L) and 1.5 µl NEBNext Ultra II End Prep Enzyme Mix (NEB, cat. no. E7546L) were mixed by pipetting and incubated at 20°C for 30 min. The reaction was inactivated by incubating at 65°C for 30 min. Subsequently, the DNA was purified with 1.8x MagMAX™ Pure Bind beads (ThermoFisher, cat. no. A58522) and the DNA was eluted at 37°C in 16 µl prewarmed (at 50°C) ddH_2O (MilliQ) for 5 min. For adapter ligation, 15 µl A-tailed DNA, 2 µl 10x T4 DNA Ligase Buffer (NEB, cat. no. B0202S), 1.5 µl T4 DNA Ligase 2,000 units/µl (NEB, cat. no. M0202M), 1 µl 10 mM ATP (NEB, cat. no. P0756S) and 0.5 µl 50 mM TruSeq Adapter (Microsynth) were mixed by pipetting and incubated at 16°C overnight. The finished ligation reaction was directly subjected to bisulfite treatment according to the manufacturer's protocol using the EZ DNA Methylation-Gold™ Kit (Zymo Research, cat. no. D5005). The DNA was eluted on the bench in 15 µl prewarmed (55°C) M-Elution Buffer (Zymo Research) for 5 min. For Polymerase Chain Reaction (PCR), 10 µl bisulfite-treated eluted DNA, 12.5 µl Q5U Master Mix (NEB, cat. no. M0597S), 1.25 µl 10 µM Truseq Forward Primer (Microsynth) and 1.25 µl 10 µM Truseq Reverse Primer (Microsynth) were mixed by pipetting and incubated at the following condition-

**Temperature**	**Time**	**Cycle**
98°C	30 sec	
98°C	15 sec	14
58°C	40 sec	
72°C	30 sec	
72°C	5 min	
16°C	∞	

Subsequently, the PCR product was purified with 0.9x AMPure XP beads (Beckman Coulter) according to the manufacturer's protocol and the DNA was eluted at 37 °C in 15 µl prewarmed (at 55°C) Elution buffer (PacBio) for 5 min. The concentration of the final library was determined using the Qubit dsDNA-HS-Assay-Kit (ThermoFisher, cat. no. Q32851) and the DNA length and quality of the final library were determined using the High Sensitivity D1000 ScreenTape assay (Agilent). Sequencing was conducted in a 100bp paired-end mode on a NexSeq1000 platform from Illumina.

Methylation calling was conducted using Bismark[8]

(https://www.bioinformatics.babraham.ac.uk/projects/bismark/) following the below provided code.

- Installation of Bismark and its dependencies

```
pip install -c bioconda bismark

pip install -c bioconda bowtie2

pip install -c bioconda samtools
```

- Reference genome preparation

Bisulfite sequencing needs your reference genome data. After place the reference genome in a directory (e.g, /path/to/genome).

```
Bismark_genome_preparation /path/to/genome
```

- Read alignment

After preparing reference genome at the previous step, our bisulfite-treated sequencing reads need to align.

```
Bismark –genome /path/to/genome -1 /data/read1-LMD-M.fastq.gz.fastqsanger.gz -2 /data/read2-LMD-M.fastq.gz.fastqsanger.gz -o /output/aligned-LMD-M.bam
```

- Methylation calling

The final step is extracted methylation information form aligned file.

```
Bismark_methylation_extractor -p --cytosine_report --CX_context --genome_folder /path/to/genome -o /output/extracted_methylation-LMD-M.bam /output/aligned-LMD-M.bam
```

Supplementary Figures

```
Tet3cd        689 TPAKKAQSEF PTCDCVEQIV EKDEGPYYTH LGSGPTVASI RELMEDRYGE KGKAIRIEKV IYTGKEGKSS
Ravichandr.       ----KAQSEF PTCDCVEQIV EKDEGPYYTH LGSGPTVASI RELMEDRYGE KGKAIRIEKV IYTGKEGKSS
Sudhamalla        TPAKKAQSEF PTCDCVEQIV EKDEGPYYTH LGSGPTVASI RELMEDRYGE KGKAIRIEKV IYTGKEGKSS
hpTet3            -------SEF PTCDCVEQIV EKDEGPYYTH LGSGPTVASI RELMEDRYGE KGKAIRIEKV IYTGKEGKSS

Tet3cd        759 RGCPIAKWVI RRHTLEEKLL CLVRHRAGHH CQNAVIVILI LAWEGIPRSL GDTLYQELTD TLRKYGNPTS
Ravichandr.       RGCPIAKWVI RRHTLEEKLL CLVRHRAGHH CQNAVIVILI LAWEGIPRSL GDTLYQELTD TLRKYGNPTS
Sudhamalla        RGCPIAKWVI RRHTLEEKLL CLVRHRAGHH CQNAVIVILI LAWEGIPRSL GDTLYQELTD TLRKYGNPTS
hpTet3            RGCPIAKWVI RRHTLEEKLL CLVRHRAGHH CQNAVIVILI LAWEGIPRSL GDTLYQELTD TLRKYGNPTS

Tet3cd        829 RRCGLNDDRT CACQGKDPNT CGASFSFGCS WSMYFNGCKY ARSKTPRKFR LTGDNPKEEE VLRNSFQDLA
Ravichandr.       RRCGLNDDRT CACQGKDPNT CGASFSFGCS WSMYFNGCKY ARSKTPRKFR LTGDNPKEEE VLRNSFQDLA
Sudhamalla        RRCGLNDDRT CACQGKDPNT CGASFSFGCS WSMYFNGCKY ARSKTPRKFR LTGDNPKEEE VLRNSFQDLA
hpTet3            RRCGLNDDRT CACQGKDPNT CGASFSFGCS WSMYFNGCKY ARSKTPRKFR LTGDNPKEEE VLRNSFQDLA

Tet3cd        899 TEVAPLYKRL APQAYQNQVT NEDVAIDCRL GLKEGRPFSG VTACMDFCAH AHKDQHNLYN GCTVVCTLTK
Ravichandr.       TEVAPLYKRL APQAYQNQVT NEDVAIDCRL GLKEGRPFSG VTACMDFCAH AHKDQHNLYN GCTVVCTLTK
Sudhamalla        TEVAPLYKRL APQAYQNQVT NEDVAIDCRL GLKEGRPFSG VTACMDFCAH AHKDQHNLYN GCTVVCTLTK
hpTet3            TEVAPLYKRL APQAYQNQVT NEDVAIDCRL GLKEGRPFSG VTACMDFCAH AHKDQHNLYN GCTVVCTLTK

Tet3cd        969 EDNRCVGQIP EDEQLHVLPL YKMASTDEFG SEENQNAKVS SGAIQVLTAF PREVRRLPEP AKSCRQRQLE
Ravichandr.       EDNRCVGQIP EDEQLHVLPL YKMASTDEFG SEENQNAKVS SGAIQVLTAF PREVRRLPEP AKSCRQRQLE
Sudhamalla        EDNRCVGQIP EDEQLHVLPL YKMASTDEFG SEENQNAKVS SGAIQVLTAF PREVRRLPEP AKSCRQRQLE
hpTet3            EDNRCVGQIP EDEQLHVLPL YKMASTDEFG SEENQNAKVS SGAIQVLTAF PREVRRLPEP AKSCRQRQLE

mTet3cd      1039 ARKAAAEKKK LQKEKLSTPE KIKQEALELA GVTTDPGLSL KGGLSQQSLK PSLKVEPQNH FSSFKYSGNA
Ravichandr.       ARKAAAEKKK LQKEKLSTPE KIKQEALELA GVTTDPGLSL KGGLSQQSLK PSLKVEPQNH FSSFKYSGNA
Sudhamalla        ARKAAAEKKK LQKEKLSTPE KI-------- ---------- ---------- ---------- ----------
hpTet3            ARKAAAEKKK ---------- ---------- ---------- ---------- ---------- ----------

mTet3cd      1109 VVESYSVLGS CRPSDPYSMS SVYSYHSRYA QPGLASVNGF HSKYTLPSFG YYGFPSSNPV FPSQFLGPSA
Ravichandr.       VVESYSVLGS CRPSDPYSMS SVYSYHSRYA QPGLASVNGF HSKYTLPSFG YYGFPSSNPV FPSQFLGPSA
Sudhamalla        ---------- ---------- ---------- ---------- ---------- ---------- ----------
hpTet3            ---------- ---------- ---------- ---------- ---------- ---------- ----------

mTet3cd      1179 WGHGGSGGSF EKKPDLHALH NSLNPAYGGA EFAELPGQAV ATDNHHPIPH HQQPAYPGPK EYLLPKVPQL
Ravichandr.       WGHGGSGGSF EKKPDLHALH NSLNPAYGGA EFAELPGQAV ATDNHHPIPH HQQPAYPGPK EYLLPKVPQL
Sudhamalla        ---------- ---------- ---------- ---------- ---------- ---------- ----------
hpTet3            ---------- ---------- ---------- ---------- ---------- ---------- ----------

mTet3cd      1249 HPASRDPSPF AQSSSCYNRS IKQEPIDPLT QAESIPRDSA KMSRTPLPEA SQNGGPSHLW GQYSGGPSMS
Ravichandr.       HPASRDPSPF AQSSSCYNRS IKQEPIDPLT QAESIPRDSA KMSRTPLPEA SQNG------ ----------
Sudhamalla        ---------- ---------- ---------- ---------- ---------- ---------- ----------
hpTet3            ---------- ---------- ---------- ---------- ---------- ---------- ----------

mTet3cd      1319 PKRTNSVGGN WGVFPPGESP TIVPDKLNSF GASCLTPSHF PESQWGLFTG EGQQSAPHAG ARLRGKPWSP
Ravichandr.       ---------- ---------- ---------- ---------- ---------- ---------- ----------
Sudhamalla        ---------- ---------- ---------- ---------- ---------- ---------- ----------
hpTet3            ---------- ---------- ---------- ---------- ---------- ---------- ----------

mTet3cd      1389 CKFGNGTSAL TGPSLTEKPW GMGTGDFNPA LGGPGFQDKL WNPVKVEEGR IPTPGANPLD KAWQAFGMPL
Ravichandr.       ---------- ---------- ---------- ---------- ---------- ---------- ----------
Sudhamalla        ---------- ---------- ---------- ---------- ---------- ---------- ----------
hpTet3            ---------- ---------- ---------- ---------- ---------- ---------- ----------

mTet3cd      1459 SSNEKLFGAL KSEEKLWDPF SLEEGTAEEP PSKGVVKEEK SGPTVEEDEE ELWSDSEHNF LDENIGGVAV
Ravichandr        ---------- ---------- ---------- ---------- ---------- ELWSDSEHNF LDENIGGVAV
Sudhamalla        ---------- ---------- ---------- ---------- -GPTVEEDEE ELWSDSEHNF LDENIGGVAV
hpTet3            ---------- ---------- ---------- ---------- ---------E ELWSDSEHNF LDENIGGVAV

mTet3cd      1529 APAHCSILIE CARRELHATT PLKKPNRCHP TRISLVFYQH KNLNQPNHGL ALWEAKMKQL AERARQRQEE
Ravichandr        APAHCSILIE CARRELHATT PLKKPNRCHP TRISLVFYQH KNLNQPNHGL ALWEAKMKQL AERARQRQEE
Sudhamalla        APAHCSILIE CARRELHATT PLKKPNRCHP TRISLVFYQH KNLNQPNHGL ALWEAKMKQL AERARQR---
hpTet3            APAHCSILIE CARRELHATT PLKKPNRCHP TRISLVFYQH KNLNQPNHGL ALWEAKMKQL AERARQRQEE

mTet3cd      1599 AARLGLGQQE AKLYGKKRKW GGAMVAEPQH KEKKGAIPTR QALAMPTDSA VTVSSYAYTK VTGPYSRWI
Ravichandr        AARLGLGQQE AKLYGKKRKW GGAMVAEPQH KEKKGAIPTR QALAMPTDSA VTVSSYAYTK VTGPYSRWI
Sudhamalla        ---------- ---------- ---------- ---------- ---------- ---------- ---------
hpTet3            AARLG----- ---------- ---------- ---------- ---------- ---------- ---------
```

Supplementary Figure S1. Sequence alignment of Tet3cd (including the LCI), hpTet3 and truncated Tet3 variants designed by M. Ravichandran *et al*. and B. Sudhamalla *et al*. Deletions in the N-terminus, LCI and C-terminus are indicated by dashed lines.

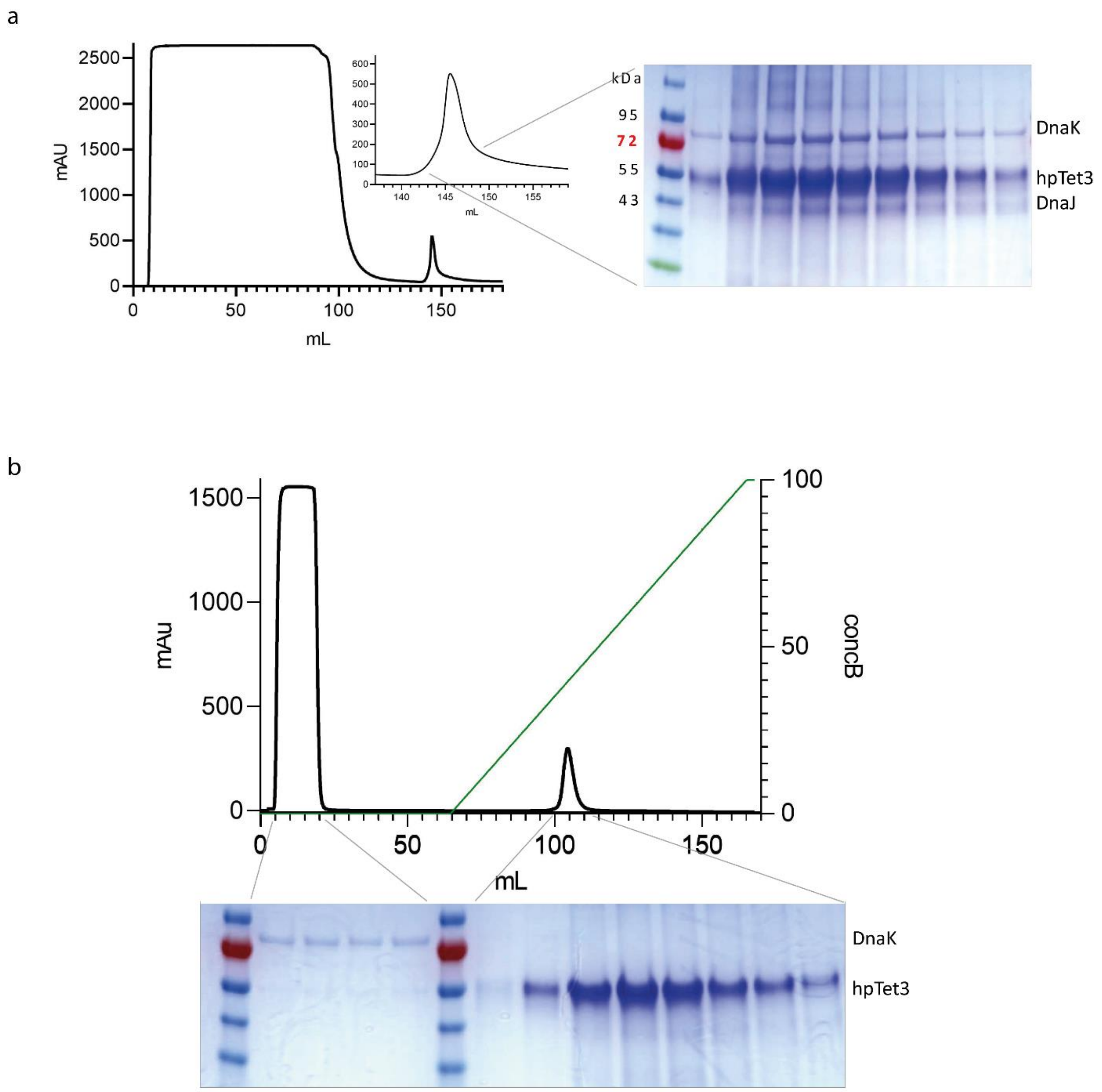

Supplementary Figure S2. Two-step purification of hpTet3 expressed in *E.coli*. N-terminally Strep(II)-tagged hpTet3 was first purified by affinity chromatography on a StrepTrap XT resin. Contaminating DnaK and DnaJ chaperons were removed from hpTet3 by cation exchange chromatography using a HiTrap Heparin HP column. Representative chromatograms and Coomassie-stained SDS-polyacrylamide gel electrophoresis analysis of the StrepTrap XT affinity chromatography elution fractions are shown in (A) and after Heparin chromatography in (B).

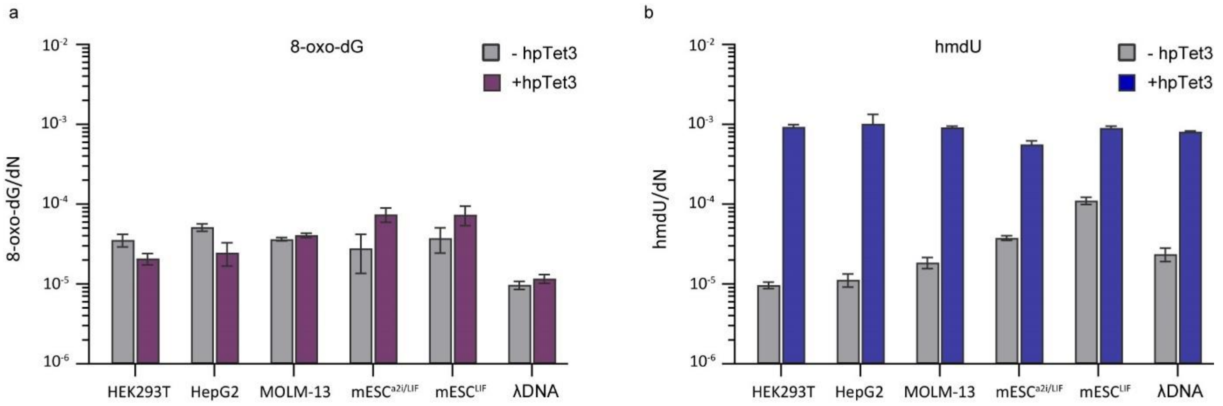

Supplementary Figure S3. 8-oxo-dG (a) and hmdU (b) levels per nucleosides (dN) of various genomic DNA (Figure 3) before and after treatment with hpTet3 as quantified by UHPLC-QQQ-MS. Depicted are mean values of biological replicates (*n*=3) with the respective ±SD.

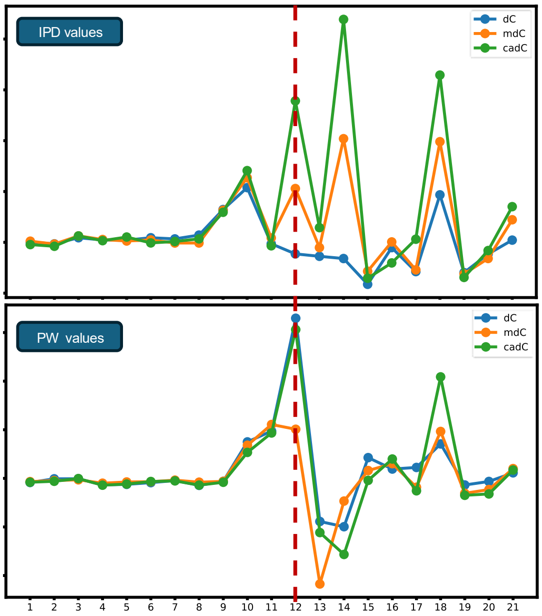

Supplementary Figure S4. The IPD and PW values extracted by ccsmeth indicate that for the model to accurately identify CpG sites, it is crucial not only to have a high IPD value but also to consider elevated PW values. As observed around position 12, the combined peak of both IPD and PW values at this point shows that the model can effectively detect a modification at this site.

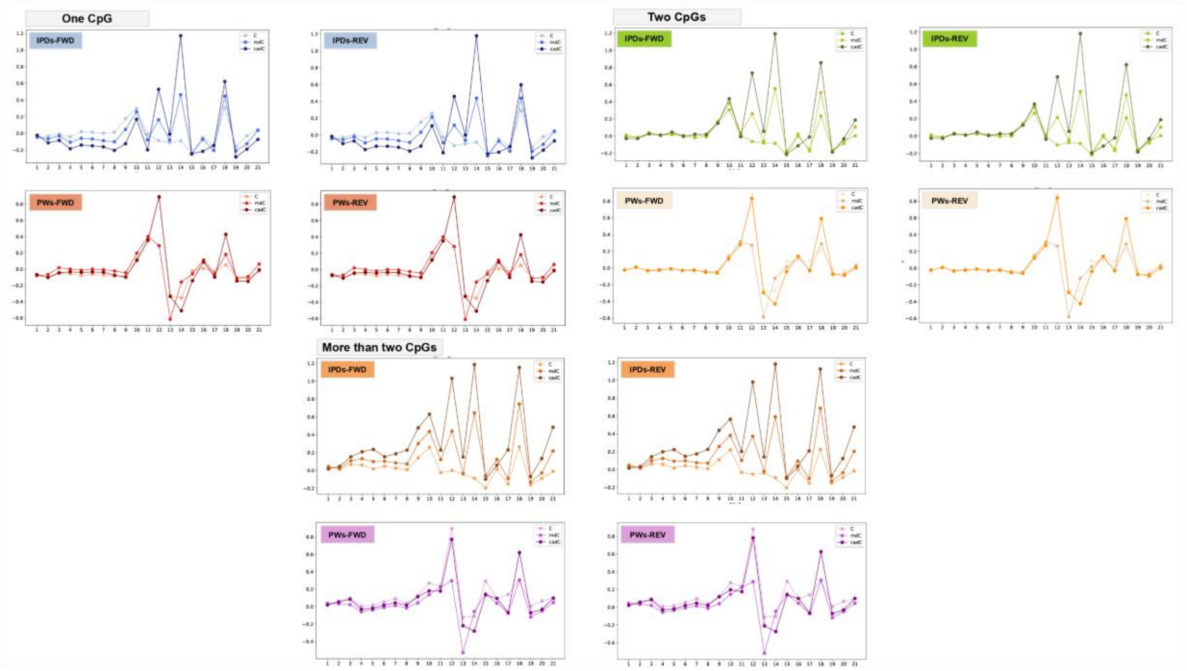

Supplementary Figure S5. IPD and PW Values for Forward and Reverse DNA Strands Across 21-mer Sequences with Varying Numbers of CpG Sites. The three subfigures illustrate the distribution of IPD (inter pulse duration) and PW (pulse width) for the 21-kmer sequences contains different numbers of CpG sites. The top-left subfigure corresponds to sequences containing exactly one CpG site, while the top-right subfigure represents sequences with two CpG sites. The bottom subfigure encompasses sequences with more than two CpG sites. Each point represents the mean IPD (or PW) value at a given k-mer position across all k-mer sequences in the given group (1-CpGs, 2-CpGs, >2CpGs). Calculations are done separately on forward (FW) and reverse (REV) IPD/PW values.

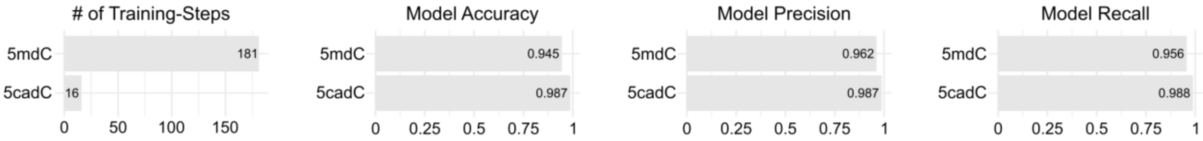

Supplementary Figure S6. Model training parameter. From left to right, number of required training steps to reach best accuracy model; achieved accuracy after training; achieved precision after training and model recall after successful training.

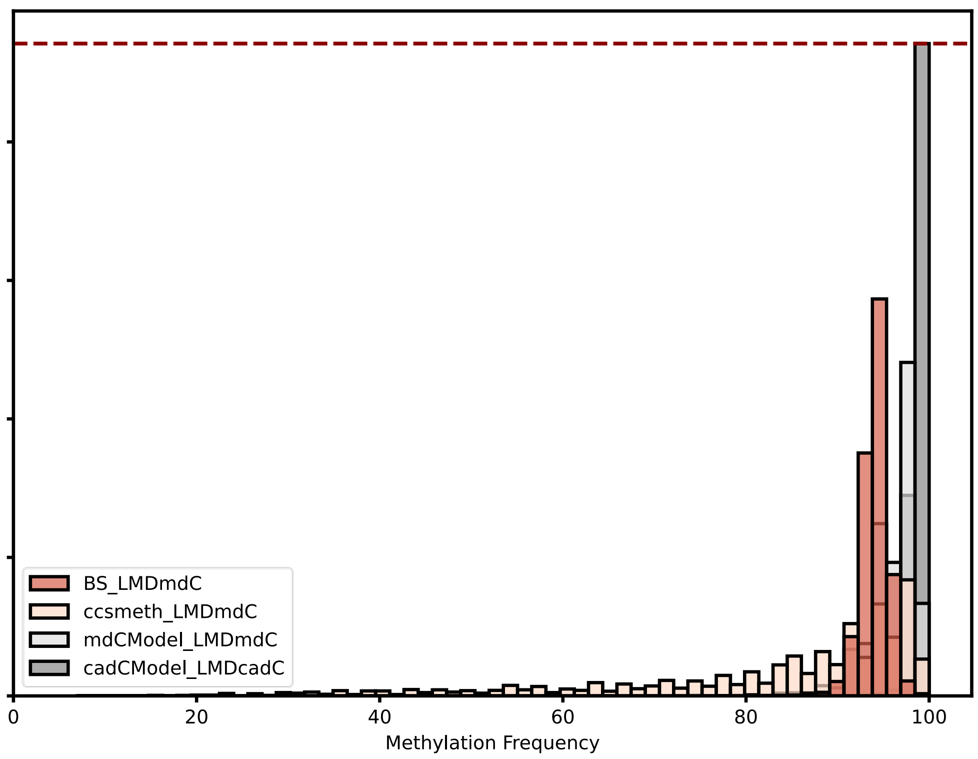

Supplementary Figure S7. Methylation frequency. The figure illustrates the methylation frequency of the lambda genome (LMD) retrieved from the distinct analysis methods/models. **BS_mdC (red),** represents the methylation frequency obtained from bisulfite sequencing of M.SssI methylated lambda DNA. **Ccsmeth_LMD_mdC (orange),** represents the methylation frequency obtained from Pacbio's c ircular consensus sequencing (CCS) of M.SssI methylated LMD using the standard ccsmeth model. **mdC-Model_LMD_mdC (light grey),** represents the represents the methylation frequency obtained from Pacbio's CCS of M.SssI methylated LMD using a ccsmeth custom trained mdC model. **cadC-Model_LMD_cadC (dark grey),** represents the methylation frequency obtained from Pacbio's CCS of M.SssI methylated and subsequently hpTet3 oxidized LMD using a ccsmeth custom trained cadC-model.

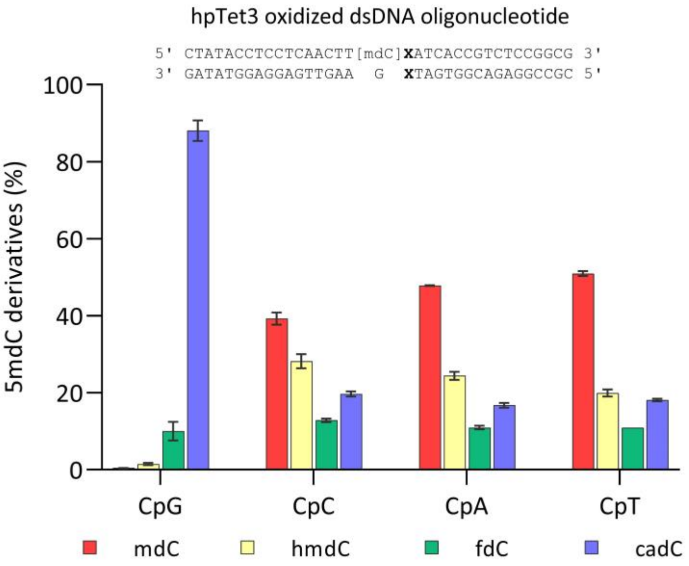

Supplementary Figure S8. UHPLC-QQQ-MS-based absolute quantification of the catalytic activity of hpTet3 on hemi-methylated dsDNA oligonucleotide containing one 5mdC in a CpG or non-CpG context under non-saturated conditions. 2 µM of the respective dsDNA was incubated with 1 µM hpTet3 for 1h at 37 °C. hpTet3 activity is dependent on the sequence context. Depicted are mean values ±s.d. of biological replicates (*n*=3).

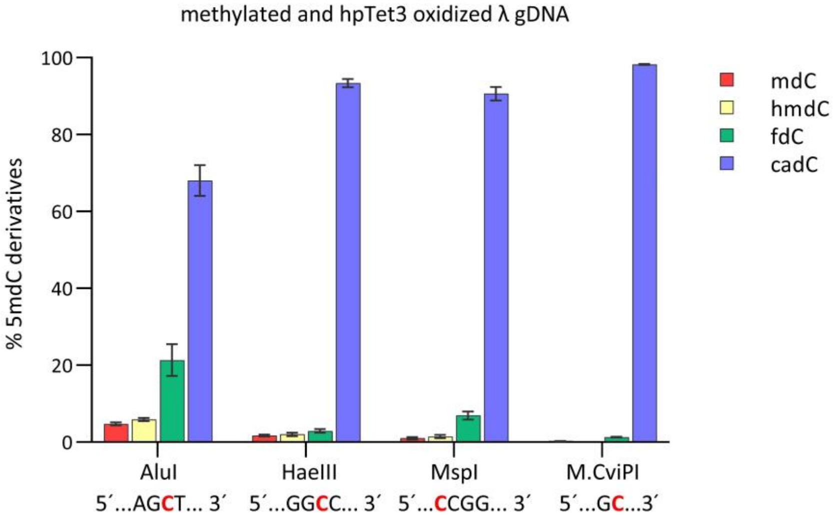

Supplementary Figure S9. UHPLC-QQQ-MS-based absolute quantification of the catalytic activity of hpTet3 on lambda DNA methylated with various methyltransferases in non-CpG context under saturated conditions. 1 µg of lambda DNA methylated with the respective methyltransferases was incubated with 15 µM (40 µg) hpTet3 for 90 min at 37 °C. Depicted are mean values ±s.d. of biological replicates (*n*=3).

Supplementary Tables

Supplementary Table S1. Oxidation of genomic DNA with hpTet3. Modified nucleosides normalized to dG are given as mean values with the respective ±SD of two independent biological replicates. M.SssI methylation efficiency of λDNA was calculated as follows: The λ genome (48,502 bp) contains 24,182 cytosine/guanine bases and 3,113 CpG dyads. A quantitative methylation would consequently result in 6,226 methylated and 17,956 unmethylated cytosines. These values were divided by the amount of total cytosine/guanine (24,182). The theoretically calculated value was compared to the obtained value after UHPLC-QQQ-MS analysis. Here, the absolute amounts of mdC and dC, as quantified by UHPLC-QQQ-MS, were once again divided by the absolute amount of dG. A methylation efficiency of 99.99% was determined by dividing the experimentally obtained value of 25.74 by the theoretical value.

methylated gDNA								
	**mdC / dG [%]**		**hmdC / dG [%]**		**fdC / dG [%]**		**cadC / dG [%]**	
	**mean**	**SD**	**mean**	**SD**	**mean**	SD	**mean**	SD
HEK293T	4.23333	0.1150	0.00693	0.000208	n.d.	n.d.	n.d.	n.d.
HepG2	4.35333	0.04509	0.001466	0.000550	n.d.	n.d.	n.d.	n.d.
MOLM-13	4.41	0.10816	0.0015	0.000794	n.d.	n.d.	n.d.	n.d.
mESC$^{a2i/LIF}$	0.81297	0.05747	0.016859	0.00153	n.d.	n.d.	n.d.	n.d.
mESC^{LIF}	2.99059	0.04278	0.04333	0.00310	n.d.	n.d.	n.d.	n.d.
M.SssI λDNA	25.74	0.00064	-	-	-	-	-	-
hpTet3 oxidized gDNA								
	**mdC / dG [%]**		**hmdC / dG [%]**		**fdC / dG [%]**		**cadC / dG [%]**	
	**mean**	**SD**	**mean**	**SD**	**mean**	**SD**	**mean**	**SD**
HEK293T	n.d.	n.d.	0.00011	7.20 x10^{-5}	0.00199	0.00014	4.23109	0.11493
HepG2	n.d.	n.d.	9.18 x10^{-5}	6.52 x10^{-5}	0.00144	0.00022	4.35176	0.04499
MOLM-13	n.d.	n.d.	9.53 x10^{-5}	4.94 x10^{-5}	0.00174	0.000626	4.40807	0.10761
mESC$^{a2i/LIF}$	n.d.	n.d.	1.40 x10^{-5}	5.51 x10^{-6}	0.000211	7.82 x10^{-5}	0.82274	0.06412
mESC^{LIF}	n.d.	n.d.	6.58 x10^{-6}	2.55 x10^{-5}	0.000825	0.00010	2.98964	0.04286
M.SssI λDNA	n.d.	n.d.	0.00051	3.60 x10^{-5}	0.00871	0.00154	25.73	0.00071
n.d. = not detected								

Supplementary Table S2 hmdU and 8-oxo-dG per dN of various genomic DNA (Figure 3) before and after treatment with hpTet3 as quantified by UHPLC-QQQ-MS.

methylated gDNA				
	**8-oxo-dG/dN**		**hmdU/dN**	
	**mean**	**SD**	**mean**	**SD**
HEK293T	3.55 x10^{-5}	6.37 x10^{-6}	9.68 x10^{-6}	8.97 x10^{-7}
HepG2	5.14 x10^{-5}	5.49 x10^{-6}	1.12 x10^{-5}	2.08 x10^{-6}
MOLM-13	3.65 x10^{-5}	1.64 x10^{-6}	1.85 x10^{-5}	2.89 x10^{-6}
mESC$^{a2i/LIF}$	2.78 x10^{-5}	1.41 x10^{-5}	3.78 x10^{-5}	2.39 x10^{-6}
mESC^{LIF}	3.75 x10^{-5}	1.31 x10^{-5}	1.11 x10^{-4}	1.19 x10^{-5}
M.SssI λDNA	9.72 x10^{-6}	1.16 x10^{-6}	2.36 x10^{-5}	4.57 x10^{-6}
hpTet3 oxidized gDNA				
HEK293T	2.08 x10^{-5}	3.39 x10^{-6}	9.28 x10^{-4}	5.90 x10^{-5}
HepG2	2.49 x10^{-5}	8.05 x10^{-6}	1.01 x10^{-3}	3.19 x10^{-4}
MOLM-13	4.07 x10^{-5}	2.64 x10^{-6}	9.15 x10^{-4}	3.38 x10^{-5}
mESC$^{a2i/LIF}$	7.46 x10^{-5}	1.51 x10^{-5}	5.60 x10^{-4}	6.15 x10^{-5}
mESC^{LIF}	7.40 x10^{-5}	1.99 x10^{-5}	9.03 x10^{-4}	4.85 x10^{-5}
M.SssI λDNA	1.16 x10^{-5}	1.41 x10^{-6}	8.09 x10^{-4}	1.79 x10^{-5}

Supplementary Table S3: The figure shows that the distribution of CpG sites with different methylation percentages across various models, as well as the WGBS results. The **BS** data on **LMD_mdC** DNA demonstrates a sparse distribution, with only 16 CpG sites falling within the 98-100% methylation range. In contrast, the **ccsmeth** and **mdC-Models** applied on **LMD_mdC** DNA show significantly higher numbers of CpG sites within this methylation range, with 329 and 963 sites, respectively. The **cadCModel** applied on **LMD_cadC** DNA, achieves 2944 CpG sites with methylation percentages in the 98-100% range. This indicates a marked improvement in the distribution and efficiency of CpG site methylation in our proposed model compared to the others.

Model	Count of different percentage of methylation				detected CpGsites	# of CpGsites in LMD
	80-90%	90-95%	95-98%	98-100%		
BS LMD_mdC	53 (~1.7%)	2315 (~74.38%)	726 (~23.32%)	16 (~0.5%)	3112	3113
Ccsmeth LMD_mdC	631 (~20.28%)	672 (~21.6%)	608 (~19.54%)	329 (~10.57%)	3111	3113
mdC-Model LMD_mdC	74 (~2.37%)	641 (~20.6%)	1425 (~45.80%)	963 (~30.95%)	3111	3113
cadC-Model LMD_cadC	1 (~0.03%)	4 (~0.12%)	162 (~5.2%)	2944 (~94.63%)	3111	3113

Supplementary Table S4: The table represents a comparative overview of the methylation percentages for 3113 CpG sites across four different models, as well as the WGBS data set. The **BS** data of **LMD_mdC** DNA has a mean methylation percentage of 94.025%, with a standard deviation (std) of 1.638%. In contrast, the **CcsMeth Model** applied to **LMD_mdC** data exhibits a lower mean methylation percentage of 83.081% and a much higher standard deviation of 17.358%. The **mdC-Model** applied to **LMD_mdC** data shows a higher mean of 95.875% with a standard deviation of 2.854%. Finally, the **cadC-Model** applied to **LMD_cadC** data demonstrates superior performance with the highest mean methylation percentage of 98.878% and the lowest standard deviation of 0.842%, indicating a very consistent methylation pattern across CpG sites. This comparative analysis underscores the efficacy of the **cadC-Model** acting on **LMD_cadC** data in achieving high and consistent methylation percentages, making it the most robust among the models evaluated.

	BS_LMD_mdC	Ccsmeth_LMD_mdC	mdCModel_LMD_mdC	cadCModel_LMD_cadC
count	3110	3110	3110	3110
mean	94.025	83.081	95.875	98.878
std	1.638	17.358	2.854	0.842
min	77.966	7	52.00	87.00
25%	93.254	77.00	95.00	99.00
50%	94.161	90.00	96.00	99.00
75%	94.951	95.00	98.00	99.00
max	99.139	100.0	100.00	100.00

Supplementary Table S5. Methylation efficiencies of the different 5mdC motifs generated by the various methyltransferases with their theoretical and calculated values.

**MTase**	**AluI**	**HaeIII**	**MspI**	**M.CviPI**			
motif in 5' -3'	AG**C**T	GG**C**C	**C**CGG	G**C**G	G**C**A	G**C**C	G**C**T
Theoretical number of methylated sites per λ genome	286	298	656	1764	2032	1630	1712
Calculated methylation efficiency**	88.83 %	90.61%	80.47%	94.2%			
Detected No. of methylated sites**	254	270	528	6724			

**Methylation efficiencies were calculated based on the quantitative data obtained by UHPLC-QQQ-MS.

Supplementary Table S6. Bisulfite (BS) sequencing of lambda gDNA methylated with GpC methyltransferase M.CviPI before and after treatment with hpTet3 under saturated conditions. 1 µg of lambda DNA methylated with M.CviPI was incubated with 15 µM (40 µg) hpTet3 for 90 min at 37 °C. The conversion of 5mdC to 5fdC and 5cadC was quantified by bisulfite sequencing. We employed M.CviPI-treated lambda DNA, which due to its GC recognition site generates a whole variety of CpH contexts (see SI Table 5). This allowed us to systematically evaluate the preference of hpTet3 in all possible +1 sequence contexts with respect to the CpH site in a 5' GCHX 3' sequence context (with X being the +1 base position). Analysis of the sequence preference of hpTet3 in the 5' G**C**HX 3' sequence context with H and X = G, C, T, A:

GpC methyltransferase M.CviPI	methylated	methylated and **oxidized** with hpTet3
Sequence context in 5'-3'	[%] methylation detected by BS	[%] methylation detected by BS
G**C**AA	88.33	0.82
G**C**AC	94.20	1.54
G**C**AG	93.77	0.77
G**C**AT	90.99	0.31
G**C**CA	93.83	0.40
G**C**CC	93.23	2.00
G**C**CG	92.69	2.13
G**C**CT	95.34	0.37
G**C**GA	93.49	0.22
G**C**GC	94.63	0.46
G**C**GG	92.00	0.30
G**C**GT	94.70	0.21
G**C**TA	94.79	1.10
G**C**TC	95.54	3.98
G**C**TG	94.01	1.82
G**C**TT	95.10	0.85

Supplementary References

[1] L. Hu, Z. Li, J. Cheng, Q. Rao, W. Gong, M. Liu, Y. G. Shi, J. Zhu, P. Wang, Y. Xu, *Cell* **2013**, *155*, 1545-1555.

[2] R. Linding, L. J. Jensen, F. Diella, P. Bork, T. J. Gibson, R. B. Russell, *Structure* **2003**, *11*, 1453-1459.

[3] D. W. A. Buchan, D. T. Jones, *Nucleic Acids Res.* **2019**, *47*, W402-W407.

[4] M. Ravichandran, D. Rafalski, C. I. Davies, O. Ortega-Recalde, X. Nan, C. R. Glanfield, A. Kotter, K. Misztal, A. H. Wang, M. Wojciechowski, M. Razew, I. M. Mayyas, O. Kardailsky, U. Schwartz, K. Zembrzycki, I. M. Morison, M. Helm, D. Weichenhan, R. Z. Jurkowska, F. Krueger, C. Plass, M. Zacharias, M. Bochtler, T. A. Hore, T. P. Jurkowski, *Sci. Adv.* **2022**, *8*, eabm2427.

[5] B. Sudhamalla, D. Dey, M. Breski, K. Islam, *Anal. Biochem.* **2017**, *534*, 28-35.

[6] F. R. Traube, S. Schiffers, K. Iwan, S. Kellner, F. Spada, M. Müller, T. Carell, *Nat. Protoc.* **2019**, *14*, 283-312.

[7] O. Y. O. Tse, P. Jiang, S. H. Cheng, W. Peng, H. Shang, J. Wong, S. L. Chan, L. C. Y. Poon, T. Y. Leung, K. C. A. Chan, R. W. K. Chiu, Y. M. D. Lo, *Proc. Natl. Acad. Sci. U. S. A.* **2021**, *118*

[8] F. Krueger, S. R. Andrews, *Bioinformatics* **2011**, *27*, 1571-1572.
